# Afro-alpine flagships revisited: Parallel adaptation, intermountain admixture and shallow genetic structuring in the giant senecios (*Dendrosenecio*)

**DOI:** 10.1371/journal.pone.0228979

**Published:** 2020-03-18

**Authors:** Felly Mugizi Tusiime, Abel Gizaw, Galina Gussarova, Sileshi Nemomissa, Magnus Popp, Catherine Aloyce Masao, Tigist Wondimu, Ahmed Abdikadir Abdi, Virginia Mirré, Vincent Muwanika, Gerald Eilu, Christian Brochmann

**Affiliations:** 1 Department of Forestry, Biodiversity and Tourism, School of Forestry, Environmental and Geographical Sciences, Makerere University, Kampala, Uganda; 2 Natural History Museum, University of Oslo, Blindern, Oslo, Norway; 3 Department of Plant Biology and Biodiversity Management, Addis Ababa University, Addis Ababa, Ethiopia; 4 Department of Botany, St Petersburg State University, St Petersburg, Russia; 5 UiT – The Arctic University of Norway, UMAK, The Arctic University Museum of Norway, Tromsø, Norway; 6 Department of Forest Biology, Sokoine University of Agriculture, Morogoro, Tanzania; 7 Botany Department, National Museums of Kenya, Nairobi, Kenya; Georg-August-Universitat Gottingen, GERMANY

## Abstract

Distantly related lineages of the enigmatic giant rosette plants of tropical alpine environments provide classical examples of convergent adaptation. For the giant senecios (*Dendrosenecio*), the endemic landmarks of the East African sky islands, it has also been suggested that parallel adaptation has been important for within-lineage differentiation. To test this hypothesis and to address potential gene flow and hybridization among the isolated sky islands, we organized field expeditions to all major mountains. We sampled all currently accepted species and all but one subspecies and genotyped 460 plants representing 109 populations. We tested whether genetic structuring corresponds to geography, as predicted by a parallel adaptation hypothesis, or to altitudinal belt and habitat rather than mountains, as predicted by a hypothesis of a single origin of adaptations. Bayesian and Neighbor-Net analyses showed that the main genetic structure is shallow and largely corresponds to geography, supporting a hypothesis of recent, rapid radiation via parallel altitude/habitat adaptation on different mountains. We also found evidence for intermountain admixture, suggesting several long-distance dispersals by wind across vast areas of unsuitable habitat. The combination of parallel adaptation, secondary contact, and hybridization may explain the complex patterns of morphological variation and the contradicting taxonomic treatments of these rare enigmatic giants, supporting the use of wide taxonomic concepts. Notably, the within-population genetic diversity was very low and calls for increased conservation efforts.

## Introduction

The enigmatic giant rosette plants of tropical alpine environments such as the giant lobelias (*Lobelia*) and the giant senecios (*Dendrosenecio*) in Africa and *Espeletia* in South America provide classical examples of convergent evolution. They are distantly related but have independently developed strikingly similar morphological, physiological and life history traits in response to the harsh tropical alpine climate with its diurnal freeze-thaw cycles [1–3]. There is no pronounced seasonal variation in temperature, but the diurnal variation is extreme at the highest altitudes ('winter every night and summer every day') [1]. Typically these giant plants have large leaf rosettes which fold up during the night to protect the buds, and they retain old leaves for insulation, accumulate large amounts of water to counteract temperature shocks, and grow taller with increasing altitude to escape the low temperatures close to the ground [1, 4, 5].

Convergent adaptation among distantly related lineages of tropical alpine plant giants is well documented [4, 6, 7], but it has also been suggested that convergent or parallel adaptation may have been important for within-lineage differentiation, for example in *Dendrosenecio* [2, 8, 9]. Parallel evolution is the repeated, independent evolution of similar traits, often reflecting adaptation caused by similar selection pressures [10]. Parallel evolution of ecotypes has been documented in a variety of organism groups, in particular along steep environmental clines which drive repeated and rapid evolutionary diversification, for example in birds (e.g. [11]), fish (e.g. [12]), and plants (e.g. [13–18]). In *Mimulus*, parallel adaptation has been documented in several traits, e.g. in flower colour [18] and pollination syndrome [19]. In *Senecio lautus*, sand dune and rocky headland ecotypes with strikingly different, genetically based morphologies and life histories have evolved numerous times during the last 200,000–500,000 years, with geographically adjacent populations being genetically most similar independently of their habitat [16, 17, 20]. There are also several examples of parallel adaptation along altitudinal gradients in mountains, e.g. in *Arabidopsis arenosa* [15], *Heliosperma pusillum* [13], and *Primula elatior* [21].

**Fig 1:**
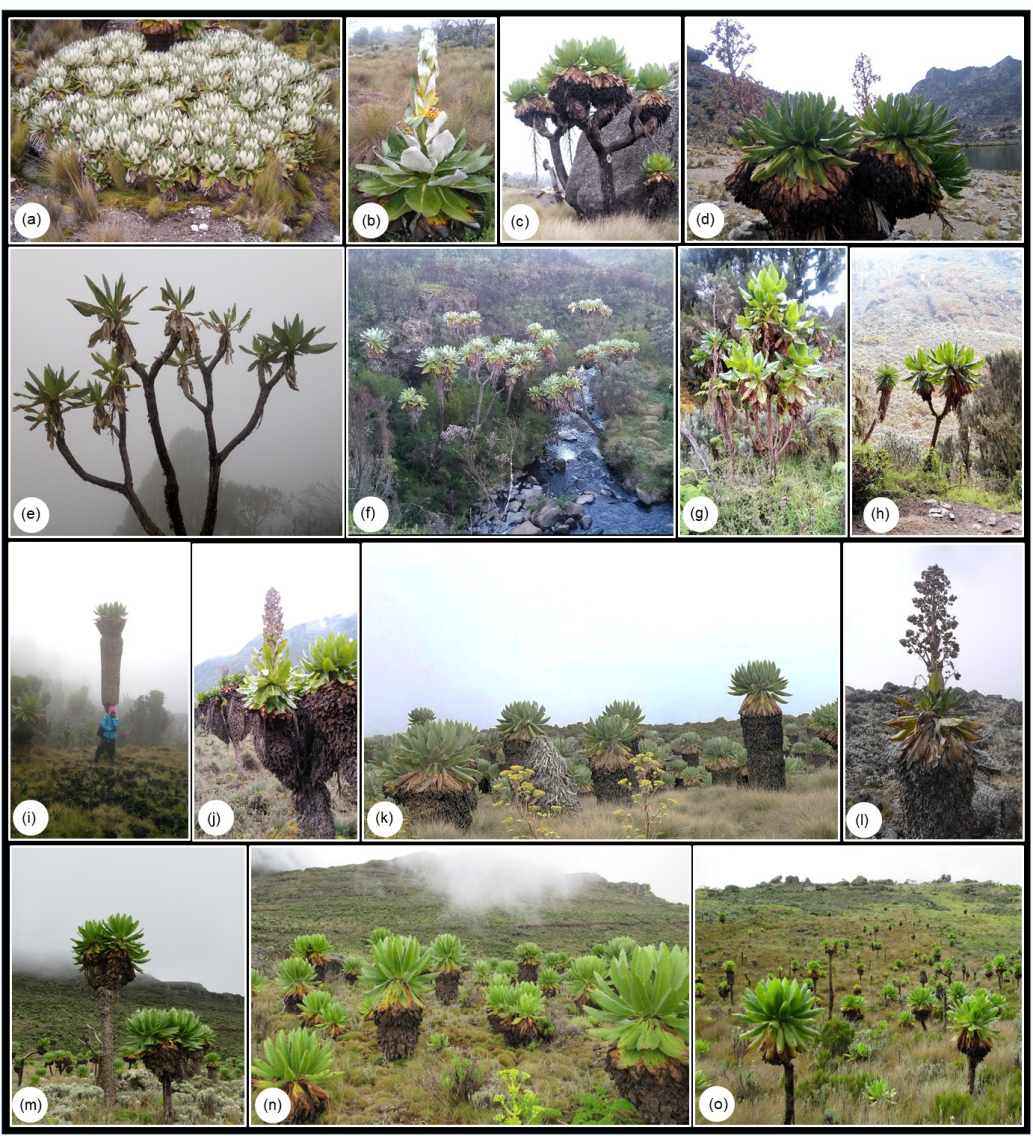
Representatives of *Dendrosenecio* illustrating the conspicuous variation in growth form and morphology in this genus (Photos: Abel Gizaw). **(a-d)**: Distinct growth form differentiation between two types of high-altitude habitats on Mt Kenya. *Dendrosenecio keniensis* (*KEN-Kn*, a & b) is a low-grown (< 1.5 m), procumbent plant that branches close to the ground and occurs on constantly water-saturated soils, whereas *D*. *keniodendron* (*KND-Kn*, c & d) is an erect giant with tall stems (up to 7 m) that branch high above the ground, occurring on well-drained soils. **(e-h)**: Plants at lower altitudes tend to have thin stems with only few old leaves kept for insulation, exemplified by plants of *D*. *battiscombei* on Mt Kenya (*BAT-Kn*, e & f) and *D*. *erici-rosenii* on Mt Ruwenzori (*ERI-Ru*, g & h). **(i-o)**: Plants at higher altitudes tend to have thick stems with a prominent layer of insulating old leaves, exemplified by plants of *D*. *adnivalis* on Mt Ruwenzori (*ADN-Ru*, i & j), *D*. *kilimanjari* on Mt Kilimanjaro (*KIL-Ki*, k & l) and *D*. *elgonensis* on Mt Elgon (*ELG-El*, m, n & o).

The giant senecios (*Dendrosenecio* (Hauman ex Hedberg) B. Nord.; Asteraceae) are along with the giant lobelias the most conspicuous and famous landmark plants of the alpine habitat in East Africa [Fig 1; 22, 23]. These giant rosette plants have woody stems that may rise 10 m above the otherwise typically low-grown alpine vegetation, and their capitula are presented in massive terminal inflorescences and produce numerous wind-dispersed achenes [1]. The giant senecios show distinct altitudinal variation in morphology [Fig 1; 8]. Most of the species and subspecies are found in the afro-alpine zone proper, i.e. above ~3500 m, but some of them also extend downwards into the transitional ericaceous zone and further into the montane forest zone. In addition, some taxa are restricted to lower altitudes, occurring in the forest zone down to 2600 m [24; Table 1]. The high-altitude plants typically have larger stems and leaves than low-altitude plants, and show stronger physiological adaptations to the harsh afro-alpine environment with their well-developed mechanisms for frost tolerance and avoidance [1, 25, 26]. On some mountains there is also more or less distinct growth form differentiation along high-altitude moisture gradients. On temporarily moist, well-drained soils, the plants tend to be erect with tall stems that branch high above the ground, whereas on constantly water-saturated soils the plants tend to be more procumbent and branch closer to the ground [8, 26].

**Table 1:** Species and subspecies of *Dendrosenecio* accepted by Knox [24] and their altitudinal and geographical range.

Species/subspecies	Altitudinal range (m)	F	E	A	Geographical range
*D*. *adnivalis* (Stapf) E.B.Knox ssp. *adnivalis*	3250–4500			X	Uganda, D. R. Congo; endemic to Mt Ruwenzori
*D*. *adnivalis* (Stapf) E.B.Knox ssp. *friesiorum* (Mildbr.) E.B.Knox	3900–4200			X	Uganda, D. R. Congo; endemic to Mt Ruwenzori
*D*. *battiscombei* (R.E.Fr. & T.C.E.Fr.) E.B.Knox	2950–4000	X	X	X	Kenya; endemic to Mt Kenya (*type locality*) and Mt Aberdare
*D*. *brassiciformis* (R.E.Fr. & T.C.E.Fr.) Mabb.	2950–3950		X	X	Kenya; endemic to Mt Aberdare
*D*. *cheranganiensis* (Cotton & Blakelock) E.B.Knox ssp. C*heranganiensis*	2600–3400	X	X		Kenya; endemic to Cherangani Hills
*D*. *cheranganiensis* (Cotton & Blakelock) E.B.Knox ssp. *dalei* E.B.Knox	3050–3500		X		Kenya; endemic to Cherangani Hills
*D*. *elgonensis* (T.C.E.Fr.) E.B.Knox ssp. *elgonensis*	2750–4200	X	X	X	Uganda, Kenya; endemic to Mt Elgon
*D*. *elgonensis* (T.C.E.Fr.) E.B.Knox ssp. *barbatipes* (Hedberg) E.B.Knox	3750–4225			X	Uganda, Kenya; endemic to Mt Elgon
*D*. *erici-rosenii* (R.E.Fr. & T.C.E.Fr.) E.B.Knox ssp. *erici-rosenii*	2750–4200	X	X	X	Uganda, Rwanda, D. R. Congo; endemic to Virunga Mts, Mt Ruwenzori, Mt Muhi, Mt Kahuzi, Mt Nyiragongo (*type locality*)
*D*. *erici-rosenii* (R.E.Fr. & T.C.E.Fr.) E.B.Knox ssp. *alticola* (Mildbr.) E.B.Knox	3400–4475			X	Uganda, Rwanda, D. R. Congo; endemic to Virunga Mts (Mt Muhavura (*neotype locality*), Mt Karisimbi, Mt Mikeno)
*D*. *johnstonii* (Oliv.) B.Nord.	2750–3350	X	X		Tanzania; endemic to Mt Kilimanjaro
*D*. *keniensis* (Baker F.) Mabb.	3300–4275			X	Kenya; endemic to Mt Kenya
*D*. *keniodendron* (R.E.Fr. & T.C.E.Fr.) B.Nord.	3650–4350			X	Kenya; endemic to Mt Kenya (*type locality*) and Mt Aberdare
*D*. *kilimanjari* (Mildbr.) E.B.Knox ssp. *cottonii* (Hutch. & G.Taylor) E.B.Knox	3600–4275			X	Tanzania; endemic to Mt Kilimanjaro
*D*. *kilimanjari* (Mildbr.) E.B.Knox ssp. *kilimanjari*	3000–3800		X		Tanzania; endemic to Mt Kilimanjaro
*D*. *meruensis* (Cotton & Blakelock) E.B.Knox	2850–3350	X	X		Tanzania; endemic to Mt Meru

The vegetation zones in which the taxa occur [24] are denoted F—montane forest zone, E—ericaceous zone and A—alpine zone

The afro-alpine ecosystem in the high mountains of East Africa represents a huge natural experiment in extreme biogeographic fragmentation. These ‘sky islands’ are separated by a ‘sea’ of unsuitable habitat: vast lower-altitude areas with savannah and semi-desert vegetation [27]. About 77% of the vascular plants species in the afro-alpine zone are endemic, indicating high level of isolation [2, 28, 29]. A central and long-debated question in African biogeography is to what degree colonization of the sky islands and subsequent intermountain gene flow does and has depended on long-distance dispersal (LDD) across unsuitable habitat, and to what extent suitable habitat corridors formed during previous cold climates and enabled gradual migration (e.g. [2, 27]). Recent ecological modelling work suggests that even during the Last Glacial Maximum (LGM), when the treeline was about 1000 m lower and the alpine habitat was about eight times larger than today, the alpine habitat in East Africa remained severely fragmented except for some closely adjacent mountain areas [30]. Thus, colonization and intermountain gene flow in alpine plants may primarily depend on rare LDD events, in the case of *Dendrosenecio* probably mediated by strong winds.

**Fig 2:**
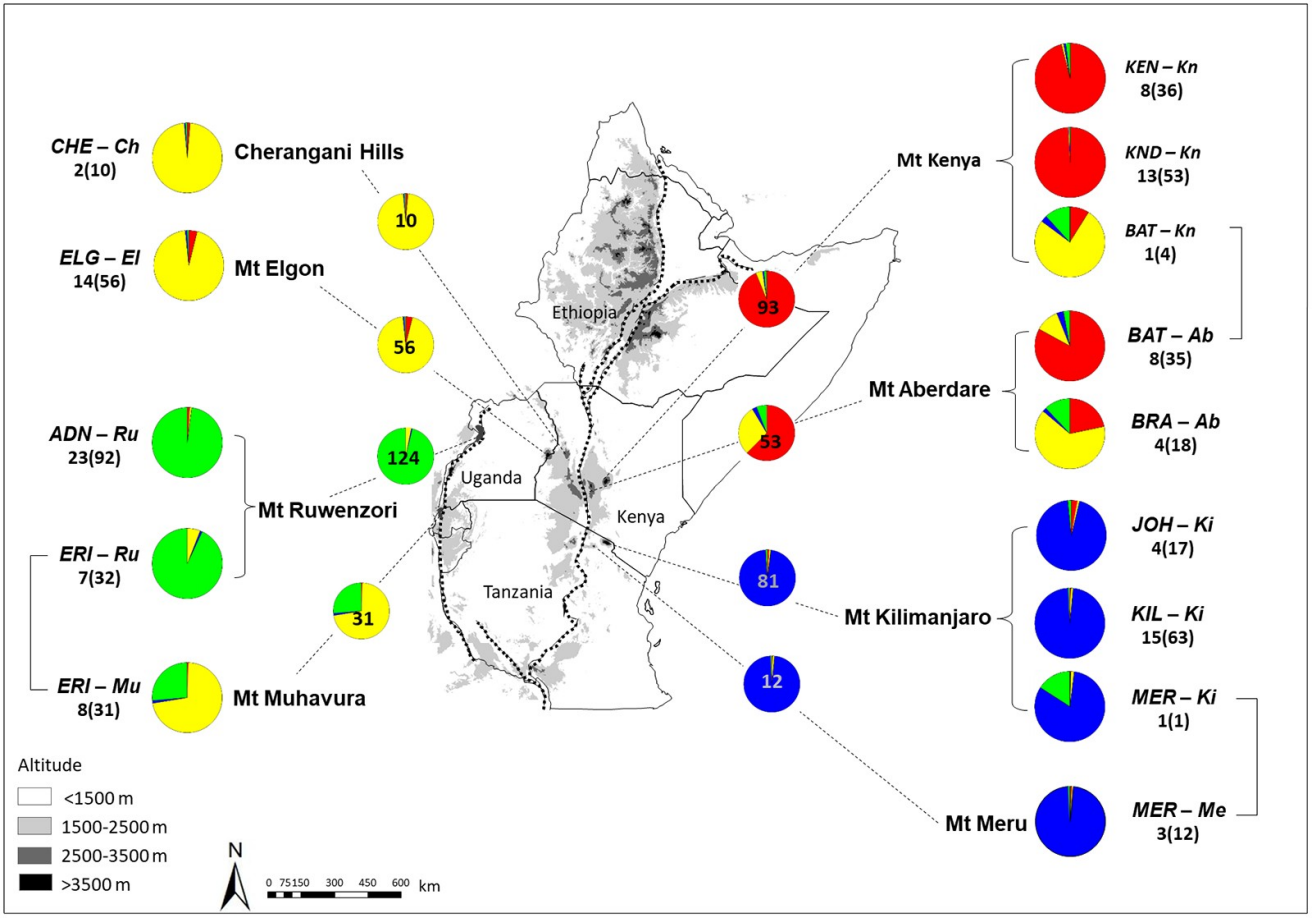
Sampling sites and main genetic structuring in *Dendrosenecio* based on the total AFLP dataset of 460 successfully analysed plants (109 populations representing all species and all but one of the subspecies accepted by Knox [24]). All species are represented with samples from all mountains from where they have been recorded, except that 1) *KND* has also been reported from Mt Aberdare, and 2) *ERI* has also been reported from some other Western Rift mountains located close to the two mountains investigated here. Colours represent the four main genetic groups inferred from STRUCTURE analyses, and the pie-charts show the proportion of admixture averaged over all individuals (corresponding to *K* = 4 in Fig 3). The pie-charts closest to the map are based on the total number of plants successfully analysed from each mountain (i.e. no taxonomic assignment). The pie-charts on the right and the left sides are based on taxonomic assignment according to morphology (*ADN*, *D*. *adnivalis; BAT*, *D*. *battiscombei; BRA*, *D*. *brassiciformis; CHE*, *D*. *cheranganiensis; ELG*, *D*. *elgonensis; ERI*, *D*. *erici-rosenii; JOH*, *D*. *johnstonii; KEN*, *D*. *keniensis; KND*, *D*. *keniodendron; KIL*, *D*. *kilimanjari; MER*, *D*. *meruensis*). The abbreviated species name is followed by abbreviated mountain name and number of populations (individual plants) successfully analysed. In three cases, plants originating from different mountains were assigned to the same species, indicated with brackets. The stippled lines represent the Great Rift Valley system.

*Dendrosenecio* is endemic to the equatorial mountains of East/Central Africa (Uganda, Kenya, Tanzania, Rwanda and the Democratic Republic of Congo, DRC;) [8]. It occurs in four distantly separated mountain groups (Fig 2; Table 1). One group is found along the western branch of the Great Rift Valley (the Western Rift Zone, WRZ, consisting of the Ruwenzori and Virunga/Muhavra mountains and some smaller mountains). The other three mountain groups are situated along the eastern branch of the Rift (the Eastern Rift Zone, ERZ), one on the western side (Mt Elgon/Cherangani Hills) and two on the eastern side (Mt Kenya/Mt Aberdare and Mt Kilimanjaro/Mt Meru).

Many species of *Dendrosenecio* have been described over the years, typically as endemic to a single mountain. Their vicarious geographical distribution and peculiar life form have inspired studies of their physiology, taxonomy and evolution for a long time [e.g. 2, 8, 22–24, 31, 32–39]. The plants show complex and mosaic-like patterns of morphological variation [8, 38], suggested to be caused by parallel adaptive radiation in different mountains and possibly coupled with sporadic long-distance dispersals, e.g. by cyclones [2]. It has also been suggested that variation in chromosome number may have caused morphological complexity [26], but all dendrosenecios are decaploid [40]. There are also many reports of putative interspecific hybrids, but mainly within single mountains [2, 8, 36, 41], and it has been proposed that some taxa could be of hybrid origin [22].

The occurrence of plants with similar morphology and physiology in similar habitats on different mountains might be explained by a single origin of adaptations on one mountain and subsequent dispersal to similar habitats on other mountains, i.e. by a niche conservatism hypothesis. This was, for example, shown to be the case for serpentine populations of *Picris hieracioides*, which were shown to have a single origin [42]. Although the evidence is quite limited, most authors favour an alternative hypothesis of independent origins of adaptations in *Dendrosenecio* on different mountains via parallel evolution. Both Hedberg [27] and Mabberley [8] believed that the high-altitude species evolved from lower-altitude montane forest ancestors. Mabberley [8] suggested that woody pachycaul forest ancestors evolved in parallel on different mountains. Another variant of the parallel adaptation hypothesis was suggested by Knox and Palmer [37], who proposed that the giant senecios originated at high altitudes on Mt Kilimanjaro within the last 1 million years, followed by dispersal to the other mountains and repeated altitudinal radiation both up and down the mountains. Their phylogenetic reconstruction was based on restriction-site variation in plastid DNA (pDNA) and suggested that the ancestry of *Dendrosenecio* is ancient and isolated within its tribe Senecioneae (see also [43]). Knox and Palmer [37] found only little pDNA variation and poor resolution within *Dendrosenecio*. This points to a recent and rapid radiation of the extant species, most likely long after the origin of the major East African mountains with the possible exception of Mt Kilimanjaro (Mt Elgon 12–23 Mya, Ruwenzori 3–8 Mya, Mt Kenya 2.5–5 Mya, Mt Kilimanjaro 1–2.5 Mya) [29, 44].

The complex morphological variation of the dendrosenecios has caused conspicuously contradicting taxonomic revisions. A large number of species were described from the 1920s to the 1960s, and Hedberg [2, 36] accepted 17 of them, mainly single-mountain endemics. In marked contrast, Mabberley [8] and Nordenstam [39] proposed to reduce the number of species to three and to recognise 10 geographic races (subspecies) and two ecological varieties. They recognised *D*. *johnstonii* as a very variable species consisting of both high- and low-altitude subspecies and, notably, occurring on all mountains. The most recent revision of *Dendrosenecio* [24] accepted 11 species and five non-autonymous subspecies and served as an initial framework for this study (Fig 2, Table 1). Eight of these 11 species occur in the afro-alpine zone (of which four are more or less restricted to this zone and three occur in all three vegetation zones), whereas the three remaining species are restricted to montane forest and ericaceous moorland [24; Fig 2, Table 1]. Notably, eight of the 11 species accepted by Knox [24] were reported as single-mountain endemics.

We carried out field sampling covering almost the entire range of *Dendrosenecio* to test the parallel adaptation hypotheses suggested by Mabberley [8] and Knox & Palmer [37], and to assess whether the taxa accepted by Knox [24] are genetically distinct. We genotyped 460 plants using Amplified Fragment Length Polymorphisms (AFLPs), which represent the entire genome and often reveal high levels of variation [45–48], and are suitable for delimiting closely related taxa and detecting admixture [49–51]. Our main objectives were: 1) To assess whether the main genetic structuring in *Dendrosenecio* corresponds to the geographic position of the mountains and mountain groups, as predicted by the parallel altitude/habitat adaptation hypothesis [8, 37], or whether the plants group according to altitudinal belt and habitat rather than mountains, as predicted by a niche conservatism hypothesis assuming a single origin of adaptations [cf. 52]. 2) To assess to what extent genetically distinct taxa can be recognized and thus provide a baseline for future morphological assessment to resolve the partly contradicting taxonomies of Hedberg [2, 36], Mabberley [8], Nordenstam [39], and Knox [24]. In addition, we used the genetic data to assess whether and to what degree admixture has occurred between mountains and mountain groups. We also explore the level of genetic diversity in these enigmatic and rare plants, which often occur in small and isolated high-altitude populations that may be threatened not only by habitat loss due to climate warming [53], but also by firewood collecting in the otherwise tree-less alpine habitat [9].

## Materials and methods

### Sampling and taxonomic assignment

We sampled 127 populations (635 individual plants) from Kenya, Uganda and Tanzania, representing all 11 species and all but one (*D*. *adnivalis* ssp. *friesiorum*) of the subspecies accepted by Knox [24] (Fig 2, Table 1, S1 Appendix). Of the three species reported from more than a single mountain, we collected *D*. *battiscombei* from both recorded mountains and *D*. *erici-rosenii* from Mt Ruwenzori and Mt Muhavura (this species is also reported from some other Western Rift mountains). We sampled *D*. *keniodendron* only from Mt Kenya (where the type locality is situated), but this species has also been reported as rare on the summit of Mt Aberdare [24]. In addition, we sampled one plant from Mt Kilimanjaro that appeared morphologically most similar to *D*. *meruensis*, which has been reported as endemic to Mt Meru. Several of the species accepted by Knox [24], especially those found on the Western Rift mountains, were difficult to distinguish based on morphology. We used the key of Knox [24], which to a large degree is based on the geographic origin of the plants.

Whenever possible, we sampled five plants separated at least by 10 m to represent a population. Leaf tissue was dried in silica gel. Three of the plants were pressed as vouchers, consisting of parts of young and fully developed leaves and (if present) parts of the inflorescence. One voucher is deposited in the National Herbarium, Addis Ababa University Ethiopia (ETH); one in the Natural History Museum, University of Oslo Norway (O); and one in the country of collection, i.e. either in the East African Herbarium, National Museum of Kenya (EA), the National Herbarium of Tanzania, Arusha (NHT), or the Makerere University Herbarium, Uganda (MHU).

### DNA extraction and AFLP fingerprinting

About 1 cm^2^ of leaf tissue from each plant was ground in 2.0 mL crushing tubes with two tungsten carbide beads for 4 min at 23 Hz in a mixer mill (MM301, Retsch^®^ GmbH & Co., Haan, Germany). Total genomic DNA was extracted using MoleStrips^™^ DNA plant kit and a GeneMole^®^ robot following the manufacturer’s protocol (Mole Genetics AS, Lysaker, Norway).

AFLP fingerprinting was performed according to Gaudeul *et al*. [54] with the following modifications: the reaction mixture for the restriction ligation was incubated for 3 h; reaction mixture volumes for the polymerase chain reaction (PCR) were reduced by 50% following Kebede *et al*. [55]; pre-selective PCR products were diluted ten times; 30 pre-PCR cycles were used instead of 25; 13 selective PCR cycles were used instead of 12; and for each sample, 2.0 μL 6-FAM, 2.0 μL VIC and 3.0 μL NED labelled selective PCR products were mixed and diluted with 14 μL ddH_2_O. We added 3.5 μL of the diluted selective PCR products to 11.7 μL HIDI (formamide) and 0.3 μL GeneScan ROX^™^ 500 internal-lane size standard (Applied Biosystems, Foster City, USA). The mixture was denatured at 95 °C for 5 min followed by cooling on ice for 10 min before loading on a 16-capillary Hitachi ABI 3130XL Genetic Analyzer (Applied Biosystems, Foster City, USA).

A primer test was conducted using 38 primer combinations on two plants from different populations for each of five species (*D*. *johnstonii*, *D*. *kilimanjari*, *D*. *adnivalis*, *D*. *elgonensis* and *D*. *keniensis*). Most of the standard 3+3 selective base primer combinations gave noisy profiles, possibly because of the high DNA content in *Dendrosenecio* [56, 57]. Primers with one additional selective base were tested and some of these gave mostly clear and scorable profiles. The 3+4 primer pairs worked relatively well compared to the 3+3 primer pairs. Three primer combinations that gave many, clearly separated and reproducible polymorphic AFLP bands were selected for final analysis: 6-FAM (*Eco*RI-ATG/*Mse*I-CTAG), VIC (*Eco*RI-ACA/*Mse*I-CTCC) and NED (*Eco*RI-AGC/*Mse*I-CTG).

All 635 samples were subjected to AFLP analysis, and 10% of them were duplicated (i.e. DNA was extracted twice and the whole AFLP procedure was repeated independently) for a reproducibility test. Selection of duplicate samples and steps taken to assess reproducibility was following Bonin [45]. The raw data were analysed using ABI prism GeneScan analysis software version 3.7 (Applied Biosystems, Foster City, USA) and imported to GeneMapper^®^ software version 4.0 (Applied Biosystems, Foster City, USA) for scoring. Off-scale and double peaks were ignored and only polymorphic markers in the size range 50–500 base pairs (bp) were scored as present (1) or absent (0). Although the selection of 3+4 primer pairs reduced the number of noisy profiles, we still had to exclude 112 samples because their profiles were too noisy for scoring. For the scorable profiles, reproducibility of the AFLP markers was calculated as the average proportion of correctly reproduced bands over duplicated samples [45]. The duplicated samples (around 10% = 63 samples) were excluded after this step. For subsequent analysis of the final AFLP dataset, the data were converted into different formats using AFLPdat R-script [58].

### AFLP data analyses

The proportion of polymorphic markers (*P*) and Nei’s gene diversity (*D*; estimated as the average proportion of pairwise differences among genotypes; [59]) were calculated using AFLPdat R-script [58]. To estimate ‘genetic rarity’ (occurrence of rare markers), ‘frequency-down-weighed marker values’ (DW) were calculated according to Schönswetter & Tribsch [60] with modification as implemented in AFLPdat R-script [58]. We also counted the number of private markers (*PM*) for each species and each identified genetic group. Similarity among AFLP multilocus phenotypes was quantified using Dice’s coefficient of similarity in NTSYSpc v.2.11a [61], and Principal Coordinate Analyses (PCoAs) were used for graphical visualization.

Genetically homogenous groups were identified using model-based Bayesian clustering with STRUCTURE version 2.3.3 [62], performed at the Lifeportal, University of Oslo (http://www.lifeportal.uio.no). We tested the “no admixture” model with uncorrelated allele frequencies as well as the “admixture” model with correlated allele frequencies. Based on the results of preliminary analyses, we selected the admixture model with correlated allele frequencies for the final analyses. The recessive allele model was used to take into account the dominant nature of the AFLP data [63]. We tested *K* = 1 to *K* = 20 with 10 replicates per *K* with a burn-in period of 10^5^ and 10^6^ iterations. We used the R-script Structure-Sum-2011 [64] to summarize the results and to obtain the optimal value of *K* based on the estimated log likelihood of the data, *L*(*K*), and on similarity among replicated runs. Structure-Sum-2011 was also used to determine the rate of change in *L*(*K*) between successive K values, to estimate the coefficient of similarity among each pair of runs and to construct diagnostic plots as suggested by Evanno *et al*. [65] to choose the optimal value of K. The average estimate of individual admixture values among the replicated runs was calculated using the program CLUMPP [66] and visualized graphically using the program DISTRUCT [67].

Genetic differentiation between populations and groups of populations was assessed using Analyses of Molecular Variance (AMOVAs) in Arlequin v.3.5 [68]. A Neighbor-Net analyses using uncorrected *P* distance was constructed among 109 populations using the program SplitsTree 4.12.6 [69] and support for branches was estimated from 1,000 bootstrap replicates using the software TREECON 1.3b [70].

## Results

The final AFLP data set consisted of 455 polymorphic markers scored in 460 plants from 109 populations representing all 11 species and all but one subspecies, after removal of 175 plants that failed to provide clear and scorable profiles. The reproducibility of the AFLP markers in the final dataset was high (97.8%).

**Fig 3:**
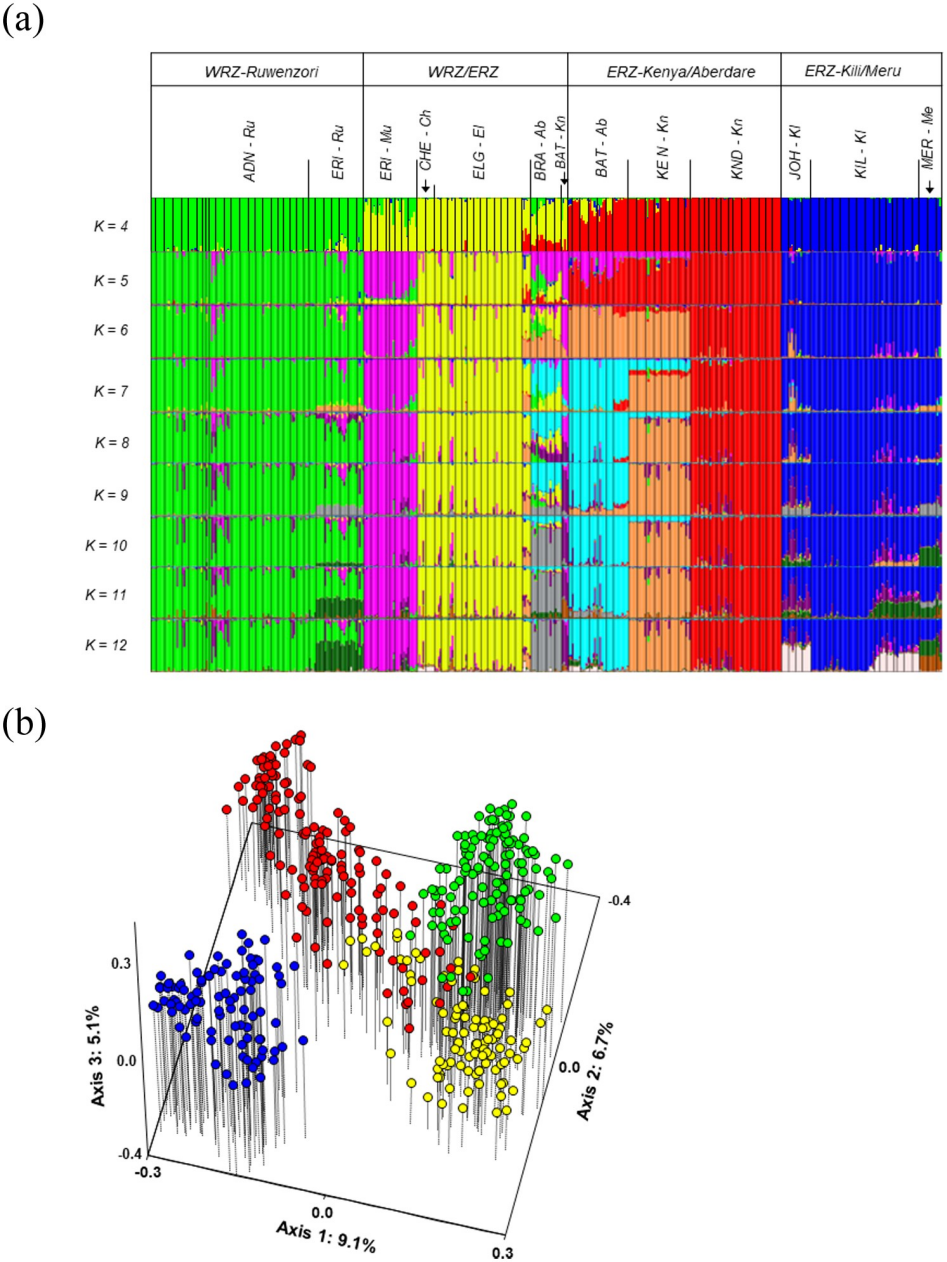
**(a)** Assignment of the 460 individual plants in the total AFLP dataset to genetic groups using STRUCTURE analyses for *K* = 4 to *K* = 12. Species and mountain names are abbreviated as in Fig 2. WRZ–Western Rift Zone mountains, ERZ–Eastern Rift Zone mountains. **(b)** Principal Coordinates Analysis (PCoA) based on Dice’s coefficient of similarity between AFLP phenotypes in the total dataset; colours show genetic groups for *K* = 4.

The STRUCTURE analyses showed gradual increment in likelihood values with increasing value of *K*, but the most consistent results among the 10 replicate runs were obtained for *K* = 4 (S1 Fig). Four main genetic groups were thus inferred to reflect the optimal partition of the data set. The four groups showed a clear geographic structure, largely corresponding to the four mountain groups (Figs 2 and 3): 1) the *WRZ-Ruwenzori group* included all plants from the WRZ mountain Ruwenzori; 2) the *WRZ/ERZ group* included all plants from the WRZ mountain Muhavura, all plants from the western side of the ERZ (Elgon and Cherangani Hills), and some of the plants from Mt Kenya and Mt Aberdare at the eastern side of the ERZ; 3) the *ERZ-Kenya/Aberdare group* included most of the plants from Mt Kenya and Mt Aberdare; and 4) the *ERZ-Kili/Meru group* included all plants from the ERZ mountains Kilimanjaro and Meru. Notably, the *WRZ/ERZ* group showed considerable admixture both with the *WRZ-Ruwenzori group* and with the *ERZ-Kenya/Aberdare group* (Figs 2 and 3a). A similar pattern was observed in the PCoA plot of the entire dataset (Fig 3b). The four groups occupied largely different parts of the PCoA plot, with the *ERZ-Kili/Meru group* as most distinct and with considerable intermixing among the other three groups.

We also assessed the results of STRUCTURE analyses for stepwise higher numbers of *K*s (Fig 3a), resulting in some additional, quite distinct genetic groups that largely corresponded to taxonomy and/or geography. Whereas no meaningful subdivision was obtained for the *WRZ-Ruwenzori* and *ERZ-Kili-Meru* groups, increasing the number of *K*s resulted in three additional genetic groups in each of the *WRZ/ERZ* and *ERZ-Kenya/Aberdare* groups (Fig 3a). In the *WRZ/ERZ group*, the WRZ plants (Muhavura) together with some plants from ERZ (Mt Kenya; *BAT-Kn*) formed a distinct group for *K* = 5, and the Aberdare plants (*BRA-Ab*) formed a distinct group around *K* = 10. The *ERZ-Kenya/Aberdare* group was subdivided into three distinct groups around *K* = 7, two containing plants from Mt Kenya and one containing Aberdare plants. Thus, we retrieved a total of eight groups that largely corresponded to taxonomy and/or geography (Fig 3a).

**Fig 4:**
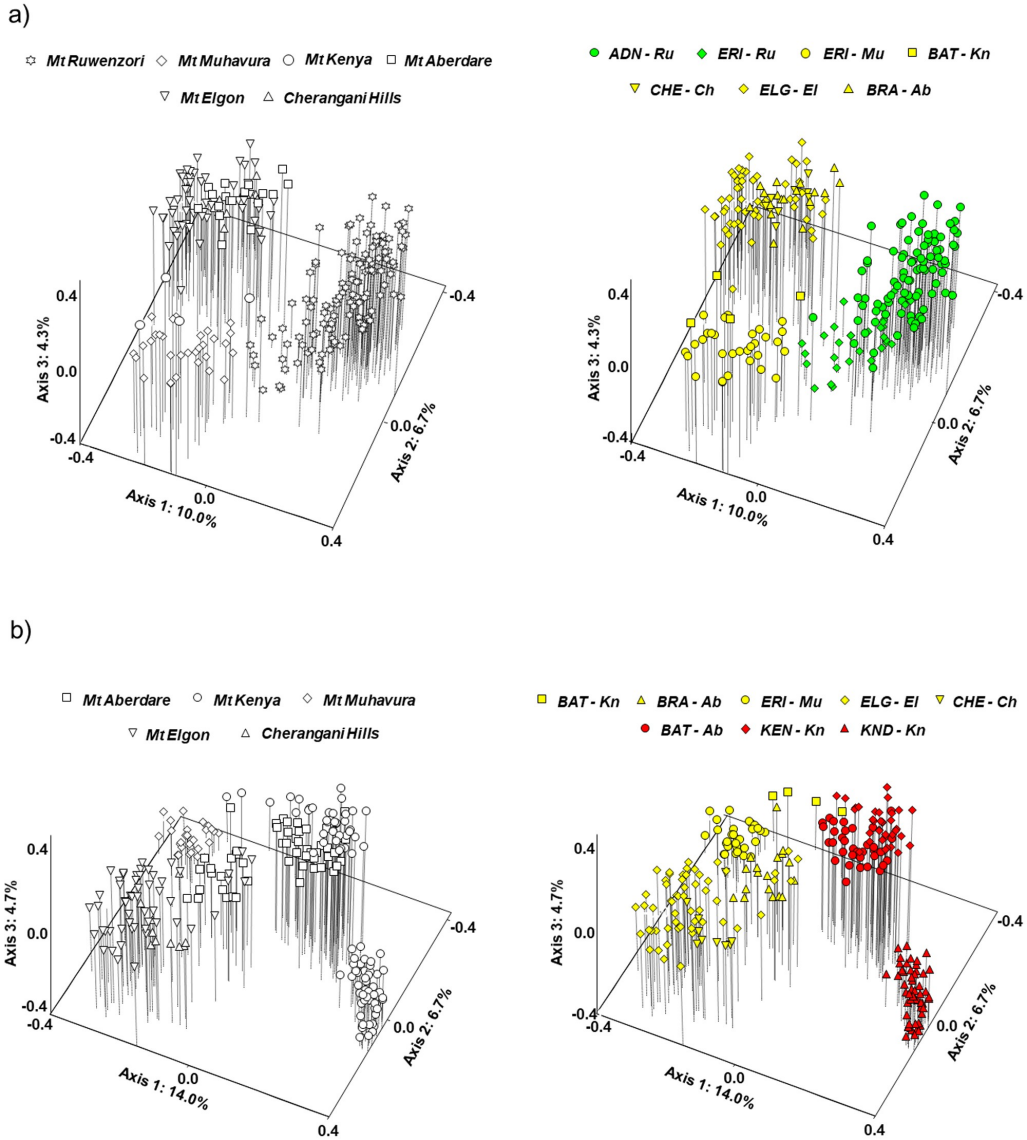
Separate principal coordinates analyses (PCoA) based on Dice’s coefficient of similarity between AFLP phenotypes for subsets of the total dataset to assess details of the admixture between three of the four main genetic groups. **(a)** The *WRZ-Ruwenzori* and *WRZ/ERZ* genetic groups; **(b)** The *ERZ-Kenya/Aberdare* and *WRZ/ERZ* genetic groups. Plots to the left show mountains, plots to the right show taxonomic annotations. Abbreviations as in Fig 2.

In a separate PCoA of the *WRZ/ERZ* group combined with the *WRZ-Ruwenzori* group (Fig 4a), the WRZ plants formed a diagonal continuum along axes 1 and 2 with the two mountains (Ruwenzori and Muhavura) placed at opposite sides. Some plants from the ERZ mountain Kenya (*BAT-Kn*; hereafter referred to as ‘the putative Mt Kenya hybrids’) grouped closely with the Mt Muhavura plants, consistent with the STRUCTURE analyses (Fig 3a). The remaining ERZ plants were fairly well separated in this PCoA.

In a separate PCoA of the *WRZ/ERZ* group combined with the *ERZ-Kenya/Aberdare* group (Fig 4b), the two genetic groups were fairly well separated except for being bridged by the putative Mt Kenya hybrids. The most distinct plants in this analysis corresponded to *D*. *keniodendron* from Mt Kenya. Notably, some of the Aberdare plants (*BRA-Ab*; hereafter referred to as ‘the putative Mt Aberdare hybrids’) grouped closely with plants from Mt Elgon.

**Fig 5:**
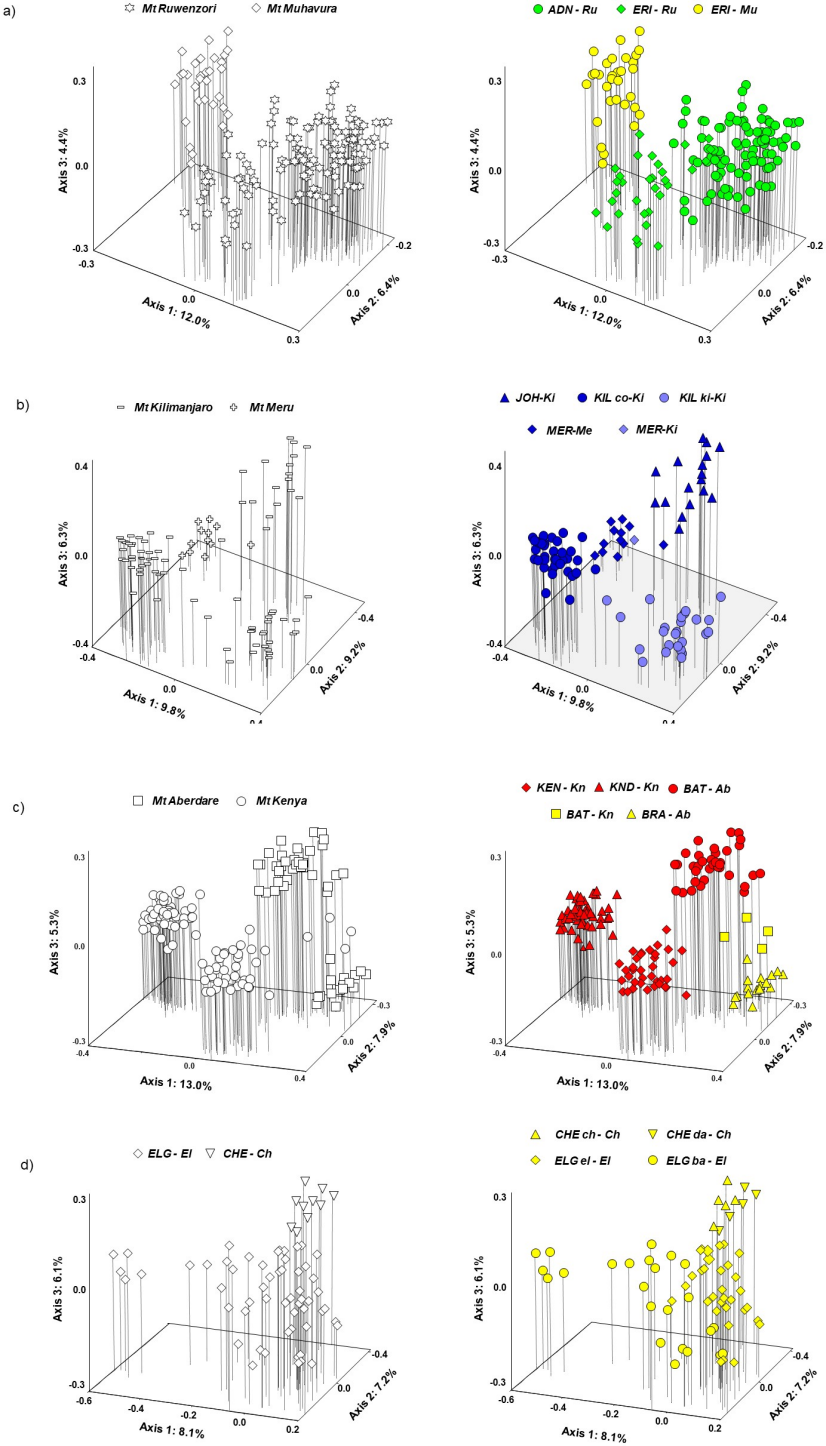
Separate principal coordinates analyses (PCoA) based on Dice’s coefficient of similarity between AFLP phenotypes for subsets of the total dataset representing each of the four main mountain groups. Plots to the left show mountains, plots to the right show taxonomic annotations. Abbreviations as in Fig 2, except in 4b where *KIL co-Ki* (*D*. *kilimanjari* ssp. *cottonii*), *KIL ki-Ki* (*D*. *kilimanjari* ssp. *kilimanjari*) and in 4d where *CHE ch-Ch* (*D*. *cheranganiensis* ssp. *cheranganiensis)*, *CHE da-Ch* (*D*. *cheranganiensis* ssp. *dalei*), *ELG el-EL* (*D*. *elgonensis* ssp. *elgonensis*) and *ELG ba-EL* (*D*. *elgonensis* ssp. *barbatipes*) as indicated in S1 Appendix.

Separate PCoAs for each of the four mountain groups revealed more or less continuous variation except for the Kenya/Aberdare group (Fig 5). In the WRZ mountain group (Fig 5a), the two mountains (Mt Ruwenzori and Mt Muhavura) formed different parts of a continuum along axis 1, bridged by the plants taxonomically assigned to *ERI-Ru*. In the Kilimanjaro/Meru mountain group (Fig 5b), the two subspecies of *D*. *kilimanjari* were fairly well separated along axis 1, whereas axes 1 and 3 more or less distinguished the Mt Kilimanjaro endemic *D*. *johnstonii* from the Mt Meru endemic *D*. *meruensis*. However, one plant collected in Mt Kilimanjaro (noted in the field as morphologically most similar to Meru plants) and one plant collected in Mt Meru were placed more or less intermediately between these two mountains in the analysis (hereafter referred to as ‘the putative Mt Kilimanjaro/Mt Meru hybrids’). In the Kenya/Aberdare mountain group (Fig 5c), three distinct groups were distinguished, corresponding to three species (*D*. *keniodendron* and *D*. *keniensis* from Mt Kenya, and *D*. *battiscombei* from Mt Aberdare), in addition to the two groups of putative hybrids (*BAT-Kn* and *BRA-Ab*). In the Elgon/Cherangani mountain group (Fig 5d), the Cherangani plants were placed at one extreme of axis 2, but the variation was continuous and the two species described as single-mountain endemics could not be distinguished, in agreement with the STRUCTURE analyses (Fig 3a).

**Fig 6:**
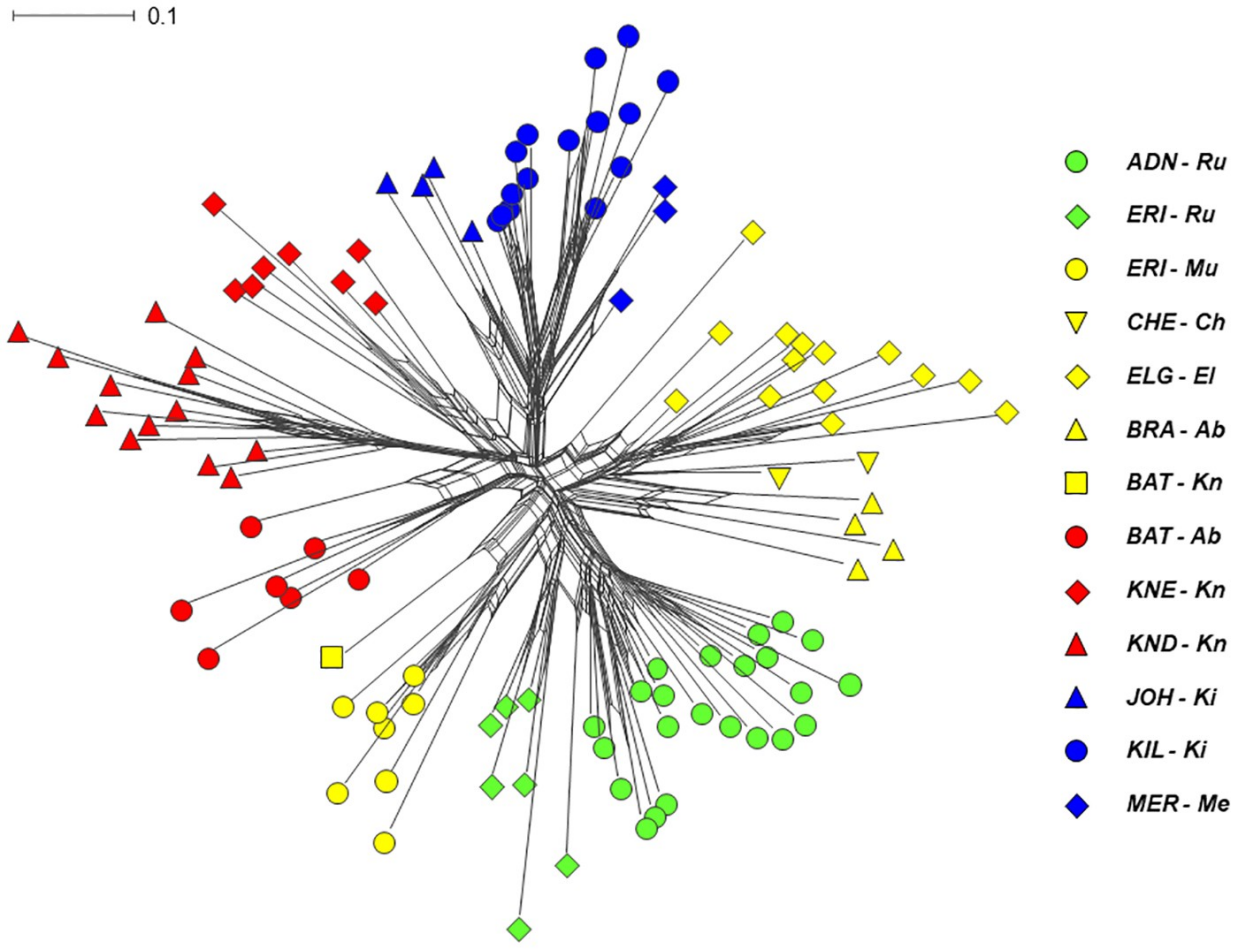
Neighbor-Net diagram computed from pairwise *F*_st_ values as measures of distance among 109 populations of *Dendrosenecio*, calculated from the total AFLP dataset comprising 460 individual plants successfully analysed for AFLP. Colours show the main genetic groups inferred in the STRUCTURE analyses. Branch supports were estimated using 1000 bootstrap replicates; no major branches obtained >50% support. Abbreviations as in Fig 2.

In the Neighbor-Net analyses (Fig 6), no major branches obtained > 50% bootstrap support. The Neighbor-Net diagram was essentially star-shaped, and the plants from the same mountain or mountain group grouped together with exception of the putative inter-mountain-group hybrids.

The average within-population gene diversity and percentage of polymorphic loci were low (Table 2). Gene diversity ranged from *D* = 0.042 ± 0.012 to *D* = 0.084 ± 0.015. Genetic rarity ranged from DW = 0.68 to DW = 2.31. The highest numbers of private markers were observed on Mt Elgon (*PM* = 33) and the lowest in *D*. *battiscombei* on Mt Kenya (*PM* = 1). The within-population genetic diversity was quite similar among the four mountain groups (*P* = 48–62%, *D* = 0.081–0.099), whereas genetic rarity was somewhat higher in Mt Elgon/Cherangani Hills (DW = 1.55) than in the other mountain groups (DW = 0.83–1.03).

**Table 2:** Genetic diversity and rarity in *Dendrosenecio* based on 455 AFLP markers scored in 460 individual plants from 109 populations, representing all 11 species accepted by Knox [24].

Groups	Species abbreviation	Country	Mountain	*n*	DW	*P* (*%*)	*D* (*sd*)	*PM*
**Genetic group**								
*WRZ-Ruwenzori*				124	0.75	52.09	0.068 (0.005)	31
*D*. *adnivalis*	*ADN*	Uganda	Mt Ruwenzori	92	0.76	45.93	0.051 (0.011)	20
*D*. *erici-rosenii*	*ERI*	Uganda	Mt Ruwenzori	32	0.71	29.67	0.045 (0.014)	6
*WRZ/ERZ*				119	1.36	72.75	0.078 (0.012)	74
*D*. *battiscombei*	*BAT*	Kenya	Mt Kenya	4	1.51	12.97	0.068	1
*D*. *brassiciformis*	*BRA*	Kenya	Mt Aberdare	18	1.02	25.93	0.050 (0.006)	4
*D*. *cheranganiensis*	*CHE*	Kenya	Cherangani Hills	10	2.00	24.18	0.071 (0.009)	6
*D*. *elgonensis*	*ELG*	Kenya	Mt Elgon	56	1.47	48.35	0.062 (0.018)	33
*D*. *erici-rosenii*	*ERI*	Uganda	Mt Muhavura	31	1.15	35.38	0.052 (0.010)	11
*ERZ-Kenya/Aberdare*				124	0.84	51.43	0.071 (0.009)	25
*D*. *battiscombei*	*BAT*	Kenya	Mt Aberdare	35	1.06	32.97	0.051 (0.014)	7
*D*. *keniodendron*	*KND*	Kenya	Mt Kenya	53	0.81	30.99	0.042 (0.012)	7
*D*. *keniensis*	*KEN*	Kenya	Mt Kenya	36	0.68	27.25	0.048 (0.009)	3
*ERZ-Kili/Meru*				93	1.03	48.35	0.074 (0.017)	37
*D*. *johnstonii*	*JOH*	Tanzania	Mt Kilimanjaro	17	0.68	19.78	0.046 (0.007)	2
*D*. *kilimanjari*	*KIL*	Tanzania	Mt Kilimanjaro	63	0.86	37.14	0.050 (0.009)	15
*D*. *meruensis*	*MER*	Tanzania	Mt Meru	13	2.31	27.47	0.084 (0.015)	13
**Mountain group**								
*Mt Kenya/Mt Aberdare*				146	0.88	59.34	0.094	39
*Mt Kilimanjaro/Mt Meru*				93	1.03	48.35	0.081	37
*Mt Ruwenzori/Mt Muhavura*				155	0.83	62.42	0.082	47
*Mt Elgon/Cherangani Hills*				66	1.55	53.63	0.099	47

Calculations were made for the four mountain groups and for the main genetic groups inferred from STRUCTURE analyses. *n*—number of individuals successfully analysed, DW—frequency down-weighed marker value as a measure of genetic rarity, *P(%)*—percentage of polymorphic loci, *D*—average intra-population diversity (standard deviation in parenthesis), *PM*—number of private markers.

In the non-hierarchical AMOVA, 47.98% of the variation was found within populations (Table 3). In hierarchical AMOVAs, 29.34% of the variation was found among the 11 currently accepted species, 19.17% among the four main mountain groups, 21.70% among the four main genetic groups, and 11.93% between the ERZ and WRZ mountains. When analysed for each mountain group, the highest proportion of variation among the currently accepted species was found for Kenya/Aberdare (27.35%). Notably, the proportion of variation found among populations was higher than that among the accepted species in all mountain groups except Kenya/Aberdare (Table 3).

**Table 3:** Non-hierarchical and hierarchical analyses of molecular variance (AMOVAs) in *Dendrosenecio* based on 455 AFLP markers scored in 460 individuals from 109 populations (representing all 11 species accepted by Knox [24]).

	Source of variation	d.f.	Variance	Percentage of variation
All populations	Among populations	108	12.69	52.02
	Within populations	351	11.70	47.98
11 species	Among species	10	7.41	29.34
	Among populations	98	6.13	24.29
	Within populations	351	11.70	46.36
Four mountain groups	Among groups	3	4.93	19.17
	Among populations	105	9.06	35.27
	Within populations	351	11.70	45.56
*Mt Kenya/Mt Aberdare*	Among species	3	6.39	27.35
	Among populations	29	6.07	26.00
	Within populations	113	10.89	46.65
*Mt Kilimanjaro/Mt Meru*	Among species	2	4.06	19.56
	Among populations	19	4.62	22.26
	Within populations	71	12.06	58.18
*Mt Ruwenzori/Mt Muhavura*	Among species	1	2.87	14.14
	Among populations	36	6.21	30.58
	Within populations	117	11.21	55.28
*Mt Elgon/Cherangani Hills*	Among species	1	2.32	9.38
	Among populations	14	8.12	32.78
	Within populations	50	14.28	57.78
Four genetic groups	Among groups	3	5.59	21.70
	Among populations	105	8.47	32.88
	Within populations	351	11.70	45.42
*WRZ-Ruwenzori group*	Among species	1	2.71	14.30
	Among populations	28	5.10	26.89
	Within populations	94	11.16	58.81
*WRZ/ERZ group*	Among species	4	5.58	21.65
	Among populations	24	7.07	27.41
	Within populations	90	13.13	50.94
*ERZ-Kenya/Aberdare group*	Among species	2	5.92	26.66
	Among populations	25	5.60	25.19
	Within populations	96	10.69	48.14
*ERZ-Kili/Meru group*	Among species	2	4.00	19.38
	Among populations	19	4.68	22.63
	Within populations	71	11.98	57.98
Eight mountains	Among mountains	7	6.61	25.92
	Among populations	101	7.20	28.21
	Within populations	351	11.70	45.87
*ERZ* vs *WRZ* mountain groups	Among groups	1	3.11	11.93
	Among populations	107	11.28	43.23
	Within populations	351	11.70	44.84

Total variance was partitioned into the four main mountain groups and the four main genetic groups inferred from STRUCTURE analyses. *WRZ*—Western Rift Zone mountains, *ERZ*—Eastern Rift Zone mountains, d.f.–degrees of freedom

## Discussion

We have shown that repeated parallel evolution, which has been demonstrated for distantly related lineages of the enigmatic giant plants of tropical alpine environments [4, 6, 7], also have played a prominent role within the single lineage of the giant senecios in the East/Central African sky islands. By genotyping field-collected plants covering virtually the entire geographic range of *Dendrosenecio* and representing all currently accepted species and all but one subspecies, we found that the main genetic structure corresponds to geography rather than habitat (Figs 2 and 3a). This result is in accordance with the prediction from the parallel altitude/habitat adaptation hypothesis proposed by Mabberley [8] and Knox and Palmer [37]. Thus, an alternative hypothesis of a single origin of adaptations on one mountain and subsequent dispersal to similar habitats on other mountains can be rejected for *Dendrosenecio*. The optimal number of genetic groups inferred from our Bayesian analyses was four and corresponded to the four distantly separated mountain groups where the dendrosenecios occur (except for a single western mountain, Muhavura; further discussed below). Whereas high-alpine plants and low-alpine/montane plants occurring on a mountain tend to be conspicuously different in morphology, they are genetically more similar to each other than to plants from other mountains. This also applies to the growth form differentiation observed along moisture gradients on some mountains (Fig 1).

In a strict sense, our genetic data show that repeated, parallel evolution resulting in similar morphologies in similar habitats has occurred on different mountains, but do not provide direct evidence for a genetic basis of the morphological differences and that they result from adaptation caused by similar selection pressures [cf. 10]. This seems however to be the most likely explanation, for example for the increasing height and size of the plants with increasing altitude and thus decreasing temperature, allowing for better frost protection of the buds and larger water volumes in the stems to buffer against temperature shocks [1, 4, 5]. Parallel adaptation along altitudinal gradients in different mountains seems to be common in plants. In *Heliosperma pusillum* s. lat., for example, rapid and parallel evolution has resulted in six pairs of morphologically and ecologically distinct, though fully inter-fertile, montane and alpine ecotypes [13]. In *Primula elatior*, locally adapted subalpine ecotypes have developed independently from foothill populations in different mountain regions [21].

It is possible that the case of *Dendrosenecio* may be quite analogous to what has been documented in the Australian *Senecio lautus*, in which sand dune and rocky headland ecotypes with strikingly different, genetically based morphologies and life histories have evolved repeatedly during the last 200,000–500,000 years [16, 17, 20]. Even more recent parallel evolution has been shown in birds. In four species of American sparrows, parallel adaptation to an extreme environment (salt marshes) has occurred after the last glaciation, resulting in recognition of four pairs of upland and salt marsh subspecies [11]. Our AFLP data cannot be directly used for dating [71], but the shallow genetic structuring and the star-shaped Neighbor-Net tree observed in *Dendrosenecio* is consistent with recent and more or less simultaneous radiation among and within the four main mountain groups. Recent radiation is also consistent with the limited pDNA variation and lack of phylogenetic resolution found by Knox & Palmer [38], who suggested that the radiation took place within the last 1 million years. Our results suggest that many of the numerous described taxa of *Dendrosenecio* cannot be distinguished or are only poorly differentiated genetically, and the genetic variation within mountains and mountain groups is often more or less continuous, consistent with recent ecoclinal or ecotypic differentiation. It seems that only in a few cases, ecotypes may be recognized morphologically as fairly distinct subspecies, and only in a single case as distinct species (Mt Kenya: the high-grown, erect drained-habitat species *D*. *keniodendron* and the procumbent moist-habitat species *D*. *keniensis*; Figs 1a and 1b, 2a and 2b, 3a and 5).

The major genetic discontinuities among the dendrosenecios are clearly found among the four, distantly separated mountain groups, reflecting overall divergence in allopatry. The *Dendrosenecio* lineage itself may have originated before most of the East/Central African mountains were formed [37, 43], but our results are consistent with those of Knox & Palmer [37] in suggesting that the extant diversity among the dendrosenecios evolved after more or less simultaneous colonization of all four mountain groups, of which Kilimanjaro/Meru is the youngest (Meru: 0.06–2 Mya, Kilimanjaro: 1–2.5 Mya; [29, 44]). Our results cannot discriminate, however, between Mabberley’s [8] hypothesis of upward colonisation and high-altitude adaptation from forest-dwelling ancestors on each mountain, and Knox and Palmer’s [37] hypothesis of initial high-altitude colonisation and adaptation on Mt Kilimanjaro followed by dispersal and adaptation both downwards and upwards on other mountains.

Our finding of more or less continuous genetic variation along altitude and among habitats on each of most mountains suggests that ecotypic or ecoclinal differentiation occurs in the presence of gene flow. This is consistent with the many reports of within-mountain putative hybrids in the taxonomic literature [2, 8, 36, 41]. Reproductive isolation among ecotypes appears to have evolved only on Mt Kenya, where populations of the drained-habitat ecotype (*D*. *keniodendron*) in many places grow closely adjacent to populations of the wet-habitat ecotype (*D*. *keniensis*) but remain genetically clearly distinct (Fig 5c).

Gene flow also seems to occur to some extent among mountains and mountain groups via rare LDD events, and this may explain the most obvious deviation (Mt Muhavura) from the main genetic pattern. Our Bayesian analyses suggest clear cases of admixture, which most likely reflects intermountain secondary contact followed by hybridization (Figs 2 and 3a). We cannot with our data exclude with certainty the possibility that the observed pattern rather is caused by a shared ancestral gene pool, but this seems less likely given that the four main genetic groups differ in the degree of inferred admixture although they likely diverged more or less at the same time. Hybridization also seems to be the most likely explanation based on the results of the PCoA and Neighbor Net analyses (Figs 4–6). Taken together, the results pointed to several major cases of intermountain gene flow. The most extreme cases involved dispersal across the vast Ugandan lowland gap (<1500 m altitude) between the Western and Eastern Rift mountains: 1) from Mt Elgon/Cherangani Hills to Mt Muhavura, and 2) from Mt Muhavura to Mt Kenya, resulting in the putative Mt Kenya hybrids (designated as *BAT-Kn*; Figs 1e and 1f, 2, 3 and 4b). Even when the alpine habitat extended 1000 m further down the mountains and covered an area eight times larger than today during the cold and dry glaciations, migration corridors with alpine habitat did not form across the Ugandan lowland gap [30], leaving long-distance dispersal as the only plausible mechanism facilitating gene flow between Mt Muhavura and Mt Elgon/Cherangani Hills (Figs 2 & 3). This is in line with several studies suggesting that LDD events, with high levels of stochasticity, have been a major driver shaping the genetic structuring in afro-alpine plants [72–76]. The two western mountains (Mt Ruwenzori and Mt Muhavura) are ecologically quite different. Whereas Mt Ruwenzori is wet all year round and dominated by boggy habitats, Mt Muhavura is relatively dry and similar to Mt Elgon. Thus, successful establishment of plants following LDD events across the Ugandan gap may have been more likely when involving Mt Muhavura, both as source and target area, than Mt Ruwenzori.

We also found evidence for admixture between the two Western Rift mountains, Muhavura and Ruwenzori (Fig 3a). Dispersal also seems to have occurred from Mt Elgon/Cherangani Hills and nearly 300 km across the eastern branch of the Rift Valley to Mt Aberdare (the putative Mt Aberdare hybrids; *BRA-Ab*). In the Mt Kenya/Aberdare group, it seems that the taxonomic situation has been obscured by the presence of one hybrid combination in each mountain, originating after long-distance dispersal from other mountain groups (Figs 3a and 5c). If these putative hybrids are disregarded, there are three distinct genetic groups in our analyses that correspond well to three described species from these mountains, two on Mt Kenya and one on Aberdare.

Thus, our genetic data suggest that the giant senecios, which have been used as a classic example of extensive ‘vicarious speciation’ in a highly fragmented system [e.g. 77], present only limited and shallowly structured variation, likely because of the recency of their radiation combined with occasional dispersal and hybridisation. Mabberley [8, 23] and Nordenstam [39] proposed to reduce the number of species to three or four, in marked contrast to the 17 species accepted by Hedberg [2], but their delineation of species did not correspond to the major genetic groups inferred in our study. Whereas they recognized one species as occurring on all mountains and 2–3 other species restricted to Mt Kenya and Mt Aberdare, we rather found a main division into four genetic groups largely corresponding to the four mountain groups (Figs 2 and 3a). In accordance with their taxonomy, however, we found distinct genetic discontinuities within the Kenya/Aberdare mountain group (Figs 3a and 5c). Based on our genetic data, it might be reasonable to recognize only seven taxonomic species of giant dendrosenecios: two on Mt Kenya and one on each of Mt Aberdare, Mt Ruwenzori, Mt Muhavura (of putative hybrid origin), Mt Elgon/Cherangani Hills, and Mt Kilimanjaro/Mt Meru. In addition, a few subspecies and interspecific hybrid combinations might be recognized.

Our findings of low within-population genetic diversity in *Dendrosenecio* (mean *D* = 0.081–0.094 for mountain groups; Table 2, S1 Appendix) are in line with recent studies of other afro-alpine plants [72, 73, 75, 76, 78, 79]. These values are much lower than those reported in a compilation of some 300 studies of vascular plants from other regions [mean D = 0.23, SD 0.08; 80]. Most likely, the low genetic diversity observed in afro-alpine plant populations is caused by bottlenecking during unfavourable climatic periods, and calls for conservation efforts to prevent reduction of their current population sizes. The combination of peculiar life forms, many endemics, and little genetic diversity testifies that the afro-alpine region is a reservoir of a unique but vulnerable diversity.

To summarize, our results support the hypothesis that the giant senecios underwent recent and rapid diversification, which involved parallel adaptation to high-alpine/low-alpine habitats and partly to wet/drained habitats in different mountain groups. Our findings suggest that all four mountain groups were more or less simultaneously colonised, and that isolation in different mountain/mountain groups has been interrupted by episodes of dispersal and hybridisation, even across vast areas of unsuitable habitat. Combined with the putatively rapid convergence in morphological traits driven by parallel adaptation to different altitudes and habitats on different mountains, these processes may have caused the complex morphological variation among the giant senecios and resulted in the conflicting taxonomic treatments. The genetic data presented here can form a base-line for re-assessment of the morphological variation among and within the mountain groups and for the taxonomy of the dendrosenecios. Our study demonstrates the difficulties facing taxonomic delimitation in an extremely fragmented, archipelago-like system characterized by recent and rapid parallel radiation followed by periods of isolation and occasional gene flow. These enigmatic plant giants thus provide a prime example on how simple biogeographical processes can work in tandem and in complex ways.

## Supporting information

S1 Appendix Geographic origin of the 109 populations (460 individual plants) of *Dendrosenecio* successfully analysed for AFLPs, and estimates of gene diversity and rarity based on 455 AFLP markers. (DOCX)

S1 Fig Summary of the STRUCTURE results used to determine the optimal number of genetic (AFLP) groups (*K*) in *Dendrosenecio*. The analyses were based on the total AFLP dataset consisting of 460 plants from 109 populations. The plot above shows the mean value of the log probability of the data, *LnP* (*D*), and the plot below shows the rate of change of the probability between runs (Delta *K*, calculated according to Evanno [65]) with *K* ranging from 1 to 20. Ten replicates were run for each *K*.
(EPS)
