# Supplementary material for: Afro-alpine flagships revisited: Parallel adaptation, intermountain admixture and shallow genetic structuring in the giant senecios (Dendrosenecio)
Source: PLoS One. 2020 Mar 18;15(3):e0228979. doi: 10.1371/journal.pone.0228979 (PMC7080232; doi:10.1371/journal.pone.0228979)
Supplement: S1 Appendix — (DOCX) [file pone.0228979.s001.docx]

Appendix 1 Geographic origin of the 109 populations (460 individual plants) of *Dendrosenecio* successfully analysed for AFLPs, and estimates of gene diversity and rarity based on 455 AFLP markers.

| Taxon/Population ID | DNA Bank ID | Country | Locality | Alt (m) | Lat | Long | *n* | DW | *P* (*%*) | *D* |
| --- | --- | --- | --- | --- | --- | --- | --- | --- | --- | --- |
|  |  |  |  |  |  |  |  |  |  |  |
| *D. adnivalis* ssp. *adnivalis* |  |  |  |  |  |  |  |  |  |  |
| UG-2305 | O-DP-40702, O-DP-40705-40706 | Uganda | Ruwenzori Mts: Upper Bigo Valley | 3560 | 0.38602 | 29.92632 | 3 | 1.83 | 5.71 | 0.038 |
| UG-2319 | O-DP-40771-40775 | Uganda | Ruwenzori Mts: Bukurungu Valley | 3800 | 0.40078 | 29.93650 | 5 | 2.12 | 9.01 | 0.040 |
| UG-2327 | O-DP-42853-42857 | Uganda | Ruwenzori Mts: Lower Bigo Valley | 3650 | 0.39647 | 29.93153 | 5 | 3.01 | 11.65 | 0.056 |
| UG-2328 | O-DP-42858-42862 | Uganda | Ruwenzori Mts: near John Matte Hut | 3450 | 0.38802 | 29.92052 | 5 | 2.01 | 12.75 | 0.062 |
| UG-2352 | O-DP-40904-40905, O-DP-40907-40908 | Uganda | Ruwenzori Mts: Mugusu Valley | 3770 | 0.39238 | 29.91698 | 4 | 1.54 | 8.35 | 0.044 |
| UG-2359 | O-DP-40937-40941 | Uganda | Ruwenzori Mts: Mugusu Valley | 3450 | 0.38802 | 29.92052 | 5 | 2.41 | 10.99 | 0.055 |
| UG-2381 | O-DP-42882, 42884, 42886 | Uganda | Ruwenzori Mts: Lower Bukurungu Valley | 3610 | 0.37687 | 29.93000 | 3 | 2.32 | 7.91 | 0.053 |
| UG-2383 | O-DP-41038, 41039 | Uganda | Ruwenzori Mts: Lower Bukurungu Valley | 3610 | 0.37687 | 29.93000 | 2 | 1.93 | 4.62 | 0.046 |
| UG-2411 | O-DP-41155, 41159 | Uganda | Ruwenzori Mts: Lower Bujuku Valley | 3870 | 0.37677 | 29.90148 | 2 | 5.26 | 7.91 | 0.079 |
| UG-2415 | O-DP-42993-42994, O-DP-42996-42997 | Uganda | Ruwenzori Mts: Lower Bujuku Valley | 3870 | 0.37677 | 29.90148 | 4 | 1.90 | 12.31 | 0.066 |
| UG-2424 | O-DP-41195-41199 | Uganda | Ruwenzori Mts: Bukurungu Valley | 3970 | 0.38488 | 29.88867 | 5 | 3.09 | 9.89 | 0.047 |
| UG-2427 | O-DP-41206-41209 | Uganda | Ruwenzori Mts: Lower Bujuku Valley | 3960 | 0.38430 | 29.88822 | 4 | 1.72 | 9.67 | 0.054 |
| UG-2445 | O-DP-41293-41297 | Uganda | Ruwenzori Mts: Bujuku | 3950 | 0.38427 | 29.88875 | 5 | 1.99 | 9.67 | 0.045 |
| UG-2446 | O-DP-41298-41302 | Uganda | Ruwenzori Mts: Bujuku | 3950 | 0.38427 | 29.88875 | 5 | 1.75 | 10.55 | 0.052 |
| UG-2468 | O-DP-41400-41403 | Uganda | Ruwenzori Mts: Lower Bujuku Valley | 3870 | 0.37580 | 29.89895 | 4 | 1.63 | 7.03 | 0.037 |
| UG-2481 | O-DP-41446-41449 | Uganda | Ruwenzori Mts: Near Mt Becker | 4050 | 0.37258 | 29.88673 | 4 | 2.27 | 8.57 | 0.048 |
| UG-2482 | O-DP-41450-41454 | Uganda | Ruwenzori Mts: Near Mt Becker | 4050 | 0.37258 | 29.88673 | 5 | 3.30 | 12.75 | 0.061 |
| UG-2496 | O-DP-41508, O-DP-41510-41512 | Uganda | Ruwenzori Mts: Albert Peak | 4150 | 0.39178 | 29.88039 | 4 | 2.41 | 11.21 | 0.060 |
| UG-2497 | O-DP-41514-41516 | Uganda | Ruwenzori Mts: Albert Peak | 4150 | 0.39178 | 29.88039 | 3 | 2.35 | 5.05 | 0.034 |
| UG-2515 | O-DP-41580-41583 | Uganda | Ruwenzori Mts: Bujuku | 4070 | 0.38555 | 29.88570 | 4 | 1.63 | 7.25 | 0.038 |
| UG-2516 | O-DP-41585-41588 | Uganda | Ruwenzori Mts: Bujuku | 4070 | 0.38555 | 29.88570 | 4 | 1.93 | 8.35 | 0.045 |
| UG-2531 | O-DP-41653-41656 | Uganda | Ruwenzori Mts: Bujuku | 3930 | 0.38228 | 29.88838 | 4 | 1.55 | 7.47 | 0.040 |
| UG-2541 | O-DP-41700, 45858, 45860 | Uganda | Ruwenzori Mts: Bujuku | 3930 | 0.38228 | 29.88838 | 3 | 3.48 | 9.89 | 0.066 |
|  |  |  |  |  |  |  |  |  |  |  |
| *D. battiscombei* |  |  |  |  |  |  |  |  |  |  |
| KN-0729 | O-DP-28544, O-DP-28546-28553 | Kenya | Aberdare Mts: Near Honi River | 3220 | -0.36140 | 36.64320 | 9 | 4.07 | 14.51 | 0.057 |
| KN-0491 | O-DP-42490, 42491 | Kenya | Aberdare Mts: Mt Kinangop area, Gura River | 3000 | -0.54697 | 36.70653 | 2 | 5.72 | 2.86 | 0.029 |
| KN-0492 | O-DP-42493-27497 | Kenya | Aberdare Mts: Mt Kinangop area | 3040 | -0.56500 | 36.70032 | 5 | 2.96 | 9.67 | 0.045 |
| KN-0554 | O-DP-27766-27770 | Kenya | Aberdare Mts: Mt Satima area | 3870 | -0.31065 | 36.63192 | 5 | 4.17 | 11.65 | 0.056 |
| KN-0556 | O-DP-27776-27780 | Kenya | Aberdare Mts: Mt Satima area | 3900 | -0.31002 | 36.63203 | 5 | 5.71 | 14.29 | 0.073 |
| KN-0559 | O-DP-27791-27795 | Kenya | Aberdare Mts: Mt Satima area | 3910 | -0.30902 | 36.63033 | 5 | 3.26 | 11.43 | 0.055 |
| KN-0654 | O-DP-28200-28202, O-DP-28204 | Kenya | Aberdare Mts: Mt Satima area | 3670 | -0.33997 | 36.65100 | 4 | 5.05 | 8.35 | 0.044 |
| KN-1081 | O-DP-36743-36746 | Kenya | Mt Kenya: Near Lake Michaelson | 4010 | -0.14430 | 37.34892 | 4 | 7.06 | 12.97 | 0.068 |
|  |  |  |  |  |  |  |  |  |  |  |
| *D. brassiciformis* |  |  |  |  |  |  |  |  |  |  |
| KN-0516 | O-DP-42215-42218 | Kenya | Aberdare Mts: Mt Satima area | 3870 | -0.31065 | 36.63192 | 4 | 8.28 | 10.77 | 0.056 |
| KN-0517 | O-DP-42219-42223 | Kenya | Aberdare Mts: Mt Satima area | 3900 | -0.31002 | 36.63203 | 5 | 5.60 | 9.45 | 0.042 |
| KN-0518 | O-DP-42224-42228 | Kenya | Aberdare Mts: Mt Satima area | 3900 | -0.30973 | 36.63125 | 5 | 5.02 | 10.55 | 0.048 |
| KN-0519 | O-DP-42230-42233 | Kenya | Aberdare Mts: Mt Satima area | 3910 | -0.30902 | 36.63033 | 4 | 7.95 | 10.33 | 0.055 |
|  |  |  |  |  |  |  |  |  |  |  |
| *D. cheranganiensis* ssp. *cheranganiensis* | |  |  |  |  |  |  |  |  |  |
| KN-0447 | O-DP-42564-42568 | Kenya | Cherangani Hills: Tululuwa | 3180 | 1.10343 | 35.43065 | 5 | 10.94 | 16.26 | 0.077 |
| *D. cheranganiensis* ssp. *dalei* |  |  |  |  |  |  |  |  |  |  |
| KN-0448 | O-DP-42569-42573 | Kenya | Cherangani Hills: Tululuwa | 3180 | 1.17697 | 35.51838 | 5 | 11.06 | 13.19 | 0.065 |
|  |  |  |  |  |  |  |  |  |  |  |
| *D. elgonensis* ssp. *elgonensis* |  |  |  |  |  |  |  |  |  |  |
| KN-0025 | O-DP-34823-34826 | Kenya | Mt Elgon: S of Mt Koitobos | 3920 | 1.10567 | 34.60183 | 4 | 5.20 | 13.41 | 0.071 |
| KN-0037 | O-DP-34862-34865 | Kenya | Mt Elgon: S of Mt Koitobos | 4220 | 1.12390 | 34.60198 | 4 | 2.90 | 5.49 | 0.030 |
| KN-0038 | O-DP-34867-34870 | Kenya | Mt Elgon: S of Mt Koitobos | 4220 | 1.12390 | 34.60198 | 4 | 5.95 | 17.58 | 0.097 |
| KN-0039 | O-DP-34872-34875 | Kenya | Mt Elgon: S of Mt Koitobos | 4220 | 1.12390 | 34.60198 | 4 | 5.21 | 11.21 | 0.060 |
| KN-0064 | O-DP-34988-34990 | Kenya | Mt Elgon: S of Mt Koitobos | 3860 | 1.10250 | 34.60583 | 3 | 3.61 | 10.55 | 0.070 |
| KN-0327 | O-DP-44426-44430 | Kenya | Mt Elgon: S of Mt Koitobos | 3630 | 1.10067 | 34.62150 | 5 | 2.83 | 9.23 | 0.043 |
|  |  |  |  |  |  |  |  |  |  |  |
| *D. elgonensis* ssp. *barbatipes* |  |  |  |  |  |  |  |  |  |  |
| KN-0084 | O-DP-35041-35044 | Kenya | Mt Elgon: Mt Koitobos | 3950 | 1.12400 | 34.59033 | 4 | 4.90 | 14.29 | 0.078 |
| KN-0132 | O-DP-35251-35254 | Kenya | Mt Elgon: Mt Koitobos | 4000 | 1.12317 | 34.60700 | 4 | 3.75 | 10.99 | 0.059 |
| KN-0150 | O-DP-35327-35330 | Kenya | Mt Elgon: Kassowai River | 3330 | 1.09145 | 34.63115 | 4 | 2.86 | 8.79 | 0.047 |
| KN-0151 | O-DP-35332-35335 | Kenya | Mt Elgon: Kassowai River | 3290 | 1.09028 | 34.63315 | 4 | 3.06 | 11.87 | 0.063 |
| KN-0152 | O-DP-35337-35340 | Kenya | Mt Elgon: Kassowai River | 3300 | 1.09057 | 34.63228 | 4 | 3.69 | 11.21 | 0.061 |
| KN-0153 | O-DP-35342-35345 | Kenya | Mt Elgon: Kassowai River | 3290 | 1.09062 | 34.63257 | 4 | 3.76 | 11.87 | 0.064 |
| KN-0169 | O-DP-35421-35424 | Kenya | Mt Elgon: Caldera | 4030 | 1.11850 | 34.58567 | 4 | 4.30 | 14.29 | 0.080 |
| KN-0179 | O-DP-35462-35465 | Kenya | Mt Elgon: Caldera | 4040 | 1.11800 | 34.58667 | 4 | 3.18 | 7.91 | 0.041 |
|  |  |  |  |  |  |  |  |  |  |  |
| *D. erici-rosenii* ssp. *erici-rosenii* |  |  |  |  |  |  |  |  |  |  |
| UG-2024 | O-DP-43070-43073 | Uganda | Virunga Mts: Mt Muhavura, along trail to summit | 3550 | -1.37628 | 29.67153 | 4 | 4.90 | 9.01 | 0.050 |
| UG-2089 | O-DP-39875-39878 | Uganda | Virunga Mts: Mt Muhavura, near 2nd Hut | 3990 | -1.38272 | 29.67798 | 4 | 4.16 | 11.43 | 0.061 |
| UG-2091 | O-DP-39884, 39886 | Uganda | Virunga Mts: Mt Muhavura, near 2nd Hut | 3700 | -1.37820 | 29.67333 | 2 | 4.03 | 5.27 | 0.053 |
| UG-2148 | O-DP-40102-40106 | Uganda | Virunga Mts: Mt Muhavura, near 2nd Hut | 3800 |  |  | 4 | 4.05 | 10.33 | 0.055 |
| UG-2177 | O-DP-40226-40229 | Uganda | Virunga Mts: Mt Muhavura, along trail to summit | 3680 | -1.37822 | 29.67325 | 4 | 2.11 | 8.35 | 0.044 |
| UG-2272 | O-DP-40574-40577 | Uganda | Rwenzori Mts: Lower Bigo Valley | 3430 |  |  | 5 | 2.77 | 5.49 | 0.026 |
| UG-2275 | O-DP-40579-40582 | Uganda | Rwenzori Mts: Lower Bigo Valley | 3420 | 0.38602 | 29.92632 | 4 | 2.67 | 5.05 | 0.027 |
| UG-2306 | O-DP-40708-40711 | Uganda | Rwenzori Mts: Upper Bigo Valley | 3580 | 0.38558 | 29.91610 | 4 | 3.33 | 8.57 | 0.045 |
| UG-2309 | O-DP-40722-40726 | Uganda | Rwenzori Mts: Near Bigo Hut | 3490 | 0.38797 | 29.91833 | 5 | 3.42 | 12.09 | 0.057 |
| UG-2318 | O-DP-40766-40770 | Uganda | Rwenzori Mts: Bukurungu Valley | 3800 | 0.40078 | 29.93650 | 5 | 2.94 | 13.19 | 0.061 |
| UG-2354 | O-DP-40914-40918 | Uganda | Rwenzori Mts: Mugusu Valley | 3770 | 0.39238 | 29.91698 | 5 | 2.52 | 9.45 | 0.044 |
| UG-2367 | O-DP-40977-40979, O-DP-40981 | Uganda | Rwenzori Mts: Lower Bukurungu Valley | 3610 | 0.37687 | 29.93000 | 4 | 3.02 | 10.77 | 0.058 |
|  |  |  |  |  |  |  |  |  |  |  |
| *D. erici-rosenii* ssp. *alticola* |  |  |  |  |  |  |  |  |  |  |
| UG-2179 | O-DP-42939-42943 | Uganda | Virunga Mts: Mt Muhavura |  |  |  | 5 | 3.19 | 8.57 | 0.039 |
| UG-2113 | O-DP-39963-39964, O-DP-39966 | Uganda | Virunga Mts: Mt Muhavura, betw. 2nd Hut and summit | 4020 | -1.38203 | 29.67673 | 3 | 3.86 | 10.55 | 0.070 |
| UG-2090 | O-DP-39879-39883 | Uganda | Virunga Mts: Mt Muhavura, summit | 4130 | -1.38293 | 29.67753 | 5 | 3.62 | 9.23 | 0.044 |
|  |  |  |  |  |  |  |  |  |  |  |
| *D. johnstonii* |  |  |  |  |  |  |  |  |  |  |
| TZ-0517 | O-DP-36895-36898 | Tanzania | Mt Kilimanjaro: Umbwe | 3190 | -3.11987 | 37.31448 | 4 | 5.02 | 7.47 | 0.041 |
| TZ-0518 | O-DP-39188-39191 | Tanzania | Mt Kilimanjaro: Umbwe | 3100 | -3.12343 | 37.31448 | 4 | 3.70 | 7.47 | 0.041 |
| TZ-0519 | O-DP-39193, O-DP-39195-39197 | Tanzania | Mt Kilimanjaro: Umbwe | 2960 | -3.12773 | 37.31117 | 4 | 6.38 | 10.33 | 0.055 |
| TZ-0520 | O-DP-39198-39202 | Tanzania | Mt Kilimanjaro: Umbwe | 3070 | -3.12457 | 37.31253 | 5 | 5.93 | 10.11 | 0.046 |
|  |  |  |  |  |  |  |  |  |  |  |
| *D. keniensis* |  |  |  |  |  |  |  |  |  |  |
| KN-0785 | O-DP-42436-42440 | Kenya | Mt Kenya: Liki River | 3720 | -0.08490 | 37.28605 | 5 | 3.57 | 9.89 | 0.048 |
| KN-0792 | O-DP-28616-28620 | Kenya | Mt Kenya: Sirimon Route | 3650 | -0.06298 | 37.29625 | 5 | 2.00 | 7.25 | 0.036 |
| KN-0813 | O-DP-42261-42265 | Kenya | Mt Kenya: Near Shipton's Camp | 4340 | -0.13950 | 37.30917 | 5 | 4.08 | 12.97 | 0.061 |
| KN-0860 | O-DP-28842-28845 | Kenya | Mt Kenya: N of Shipton's Camp | 4230 | -0.13920 | 37.31432 | 4 | 4.05 | 10.55 | 0.057 |
| KN-0879 | O-DP-28932-28936 | Kenya | Mt Kenya: NE of Batian Peak | 4050 | -0.12138 | 37.29563 | 5 | 3.56 | 10.33 | 0.049 |
| KN-0892 | O-DP-28981-28985 | Kenya | Mt Kenya: NE of Batian Peak | 4040 | -0.12142 | 37.29563 | 5 | 2.49 | 7.47 | 0.034 |
| KN-0938 | O-DP-29171-29174 | Kenya | Mt Kenya: Shipton's Cave | 4190 | -0.13358 | 37.27650 | 4 | 4.44 | 8.57 | 0.046 |
| KN-1004 | O-DP-36511-36513 | Kenya | Mt Kenya: below Pilar | 4380 | -0.15033 | 37.33095 | 3 | 3.86 | 7.69 | 0.051 |
|  |  |  |  |  |  |  |  |  |  |  |
| *D. keniodendron* |  |  |  |  |  |  |  |  |  |  |
| KN-0781 | O-DP-28581-28585 | Kenya | Mt Kenya: Above Old Moses Camp | 3700 | -0.06762 | 37.29780 | 5 | 2.06 | 6.81 | 0.033 |
| KN-0811 | O-DP-28679-28680, O-DP-42255 | Kenya | Mt Kenya: Near Shipton's Camp | 4340 | -0.13950 | 37.30917 | 3 | 3.39 | 8.79 | 0.059 |
| KN-0823 | O-DP-42276, 42277, 42279 | Kenya | Mt Kenya: Near Shipton's Camp | 4270 | -0.14160 | 37.31392 | 3 | 2.21 | 6.81 | 0.045 |
| KN-0849 | O-DP-28793-28796, | Kenya | Mt Kenya: N of Shipton's Camp | 4230 | -0.13920 | 37.31432 | 4 | 3.09 | 9.67 | 0.052 |
| KN-0883 | O-DP-28953, O-DP-28955-28956 | Kenya | Mt Kenya: NE of Batian Peak | 4050 | -0.12138 | 37.29563 | 3 | 2.56 | 7.69 | 0.051 |
| KN-0894 | O-DP-28993-28996 | Kenya | Mt Kenya: NE of Batian Peak | 4040 | -0.12142 | 37.29563 | 4 | 2.82 | 9.89 | 0.052 |
| KN-0925 | O-DP-29110-29112 | Kenya | Mt Kenya: Shipton's Cave | 4190 | -0.13358 | 37.27650 | 3 | 3.72 | 5.71 | 0.038 |
| KN-0956 | O-DP-29252-29256 | Kenya | Mt Kenya: SE of Point Lenana | 4370 | -0.14738 | 37.33157 | 5 | 2.72 | 8.79 | 0.041 |
| KN-0977 | O-DP-36412-36416 | Kenya | Mt Kenya: SE of Point Lenana | 4390 | -0.14855 | 37.33212 | 5 | 2.14 | 6.81 | 0.032 |
| KN-1001 | O-DP-36495-36499 | Kenya | Mt Kenya: below Pilar | 4380 | -0.15033 | 37.33095 | 5 | 2.96 | 10.55 | 0.047 |
| KN-1019 | O-DP-36565-36568 | Kenya | Mt Kenya | 4050 | -0.15000 | 37.31667 | 4 | 2.02 | 3.52 | 0.018 |
| KN-1048 | O-DP-36684-36687 | Kenya | Mt Kenya | 4020 | -0.14612 | 37.34797 | 4 | 2.33 | 4.62 | 0.026 |
| KN-1097 | O-DP-36808-36812 | Kenya | Mt Kenya: Teleki Valley | 4120 | -0.16930 | 37.27533 | 5 | 2.97 | 10.99 | 0.054 |
|  |  |  |  |  |  |  |  |  |  |  |
| *D. kilimanjari* ssp. *cottonii* |  |  |  |  |  |  |  |  |  |  |
| TZ-0165 | O-DP-42624-42628 | Tanzania | Mt Kilimanjaro: Barranco | 4160 | -3.08622 | 37.32340 | 5 | 2.54 | 8.35 | 0.039 |
| TZ-0172 | O-DP-37609-37610, O-DP-37612-37613 | Tanzania | Mt Kilimanjaro: Shira Plateau | 4390 | -3.08168 | 37.32348 | 4 | 3.33 | 8.35 | 0.046 |
| TZ-0199 | O-DP-37743, 37744, 37746, 37747 | Tanzania | Mt Kilimanjaro: Karanga | 4190 | -3.09720 | 37.33555 | 4 | 2.53 | 9.89 | 0.054 |
| TZ-0200 | O-DP-42752-42755 | Tanzania | Mt Kilimanjaro: Barranco | 3900 | -3.06278 | 37.27817 | 4 | 3.10 | 12.09 | 0.067 |
| TZ-0279 | O-DP-38092-38096 | Tanzania | Mt Kilimanjaro: Mawenzi | 3820 | -3.14667 | 37.44200 | 5 | 2.27 | 9.45 | 0.044 |
| TZ-0287 | O-DP-42672-42673, O-DP-38103-38105 | Tanzania | Mt Kilimanjaro: Horombo | 3820 | -3.13500 | 37.43367 | 5 | 2.23 | 7.69 | 0.036 |
| TZ-0296 | O-DP-38140-38144 | Tanzania | Mt Kilimanjaro: Horombo | 3820 | -3.13500 | 37.43367 | 5 | 2.35 | 8.57 | 0.038 |
| TZ-0331 | O-DP-38298-38300, O-DP-42693-42694 | Tanzania | Mt Kilimanjaro: Horombo | 3860 | -3.13418 | 37.43337 | 5 | 2.52 | 10.33 | 0.050 |
|  |  |  |  |  |  |  |  |  |  |  |
| *D. kilimanjari* ssp. *kilimanjari* |  |  |  |  |  |  |  |  |  |  |
| TZ-0343 | O-DP-38339-38340, O-DP-38342 | Tanzania | Mt Kilimanjaro: Betw. Horombo Hut and Mandara Hut, 4 km from Horombo | 3290 | -3.15080 | 37.47590 | 3 | 2.66 | 8.13 | 0.054 |
| TZ-0344 | O-DP-38343-38345, O-DP-38347 | Tanzania | Mt Kilimanjaro: Betw. Horombo Hut and Mandara Hut, 4 km from Horombo | 3600 | -3.14088 | 37.45397 | 4 | 3.28 | 11.21 | 0.059 |
| TZ-0345 | O-DP-38348-38350, O-DP-38352 | Tanzania | Mt Kilimanjaro: Betw. Horombo Hut and Mandara Hut, 4 km from Horombo | 3610 | -3.14025 | 37.45263 | 4 | 2.99 | 10.11 | 0.056 |
| TZ-0534 | O-DP-45563, 45564 | Tanzania | Mt Kilimanjaro: Umbwe | 3320 | -3.11717 | 37.31448 | 2 | 3.14 | 5.49 | 0.055 |
| TZ-0535 | O-DP-45566-45569 | Tanzania | Mt Kilimanjaro: Umbwe | 3490 | -3.11332 | 37.31755 | 4 | 1.95 | 7.91 | 0.043 |
| TZ-0536 | O-DP-39203-39207 | Tanzania | Mt Kilimanjaro: Umbwe | 3420 | -3.11505 | 37.31627 | 5 | 2.24 | 9.23 | 0.045 |
| TZ-0537 | O-DP-45570-45573 | Tanzania | Mt Kilimanjaro: Umbwe | 3340 | -3.11670 | 37.31447 | 4 | 3.81 | 11.43 | 0.061 |
|  |  |  |  |  |  |  |  |  |  |  |
| *D. meruensis* |  |  |  |  |  |  |  |  |  |  |
| TZ-0491 | O-DP-42828, O-DP-39065-39068 | Tanzania | Mt Meru: Betw. Saddle Hut and Miriakamba Hut | 3610 | -3.22358 | 36.78597 | 5 | 8.19 | 15.16 | 0.071 |
| TZ-0493 | O-DP-44464-44468 | Tanzania | Mt Meru: Betw. Saddle Hut and Miriakamba Hut | 3120 | -3.22440 | 36.78505 | 5 | 10.70 | 17.36 | 0.081 |
| TZ-0510 | O-DP-39153, 39154 | Tanzania | Mt Meru: Betw. Saddle Hut and Miriakamba Hut | 3320 | -3.22035 | 36.78022 | 2 | 10.77 | 9.45 | 0.095 |
| TZ-0538 | O-DP-45575 | Tanzania | Mt Kilimanjaro: Umbwe | 3360 | -3.11637 | 37.31497 | 1 |  |  |  |

The samples represent all species and all but one subspecies accepted by Knox (2005). *n -* number of individual plants analysed, DW *-* frequency down-weighed marker value as a measure of genetic rarity, *P(%) -* percentage of polymorphic loci, *D -* within-population genetic diversity. The DNA Bank ID refers to the unique identification number in the DNA Bank at the Natural History Museum, University of Oslo (O), Norway
